# Photon Entanglement Through Brain Tissue

**DOI:** 10.1038/srep37714

**Published:** 2016-12-20

**Authors:** Lingyan Shi, Enrique J. Galvez, Robert R. Alfano

**Affiliations:** 1 Institute for Ultrafast Spectroscopy and Lasers, Department of Physics, The City College of New York, New York, NY 10031, USA; 2 Department of Biology, The City College of New York, New York, NY, 10031, USA; 3 Deparment of Chemistry, Columbia University, New York, NY, 10027, USA; 4 Department of Physics and Astronomy, Colgate University, Hamilton, NY, 13346, USA

## Abstract

Photon entanglement, the cornerstone of quantum correlations, provides a level of coherence that is not present in classical correlations. Harnessing it by study of its passage through organic matter may offer new possibilities for medical diagnosis technique. In this work, we study the preservation of photon entanglement in polarization, created by spontaneous parametric down-conversion, after one entangled photon propagates through multiphoton-scattering brain tissue slices with different thickness. The Tangle-Entropy (TS) plots show the strong preservation of entanglement of photons propagating in brain tissue. By spatially filtering the ballistic scattering of an entangled photon, we find that its polarization entanglement is preserved and non-locally correlated with its twin in the TS plots. The degree of entanglement correlates better with structure and water content than with sample thickness.

The propagation of photons through scattering media is one of the most salient aspects of optical science^1^,^2^,^3^,^4^. The light experiences many deleterious effects due to multiple scattering off the constituent particles in the media. The direction, path, phase, and polarization of a photon are changed when it travels in a scattering medium. The photon beam can break up into ballistic, snake, and diffusive components depending on the scattering mean free length, transport length, and anisotropic factor that are determined by particle size and structure^1^,^2^,^3^,^4^,^5^,^6^. When a photon enters a dense medium, such as brain slab, it is influenced by medium’s index of refraction dispersion, becoming itself a quantum quasi-particle. Quasi-particles involved in the coherent interactions with photons have fast dephasing time, e.g. 100 fs in water, 2 ps in solids in diamond, excitations at the 10 ps level, and non-radiative relaxation at the 1 ps level.

Medical diagnosis has employed a number of quantum physics-based technologies, such as optical spectroscopy, magnetic resonance imaging, and computerized tomography for understanding biological structure and functions and for disease treatment. These have been harnessed by our deep understanding of atomic and molecular energy levels, particle quantum properties such as position, spin, polarization, and their interaction with electromagnetic radiation.

The last few decades have seen the rise of the study of another quantum physics phenomenon: quantum entanglement. Beyond the fundamental questions that it generates, such as nonlocality and realism^7^,^8^, quantum entanglement provides the ultimate level of coherence between correlated particles, which can evolve coherently among entangled particles traveling separate paths and affect each other in the correlation when one of them is measured. The harnessing of the quantum entanglement has been at the core of a new type of technology known as quantum information^9^. Measurement of the exquisite level of coherence between entangled particles as they propagate through biological media could provide new medical diagnosis information not available by other methods. The method goes beyond sending single photons, let alone the coherent state of a laser, through a sample, where polarimetry and modal analysis provides information. The polarization entanglement of two photons entails a larger Hilbert space, and therefore more information. Two measures of the state of the pair are tangle (T) and linear entropy (S). They characterize distinct aspects of the state: with non-separability quantified by T and coherence by S. When one of the twin photons enters a scattering turbid medium, such as a sample with beads or a tissue, the initial entanglement of the input photon will in general be degraded by multiple scattering. The phase and polarization coherence of the pair will degrade in general to a mixed state, reducing T and increasing S of the state shared with the other twin photon. How does the change in these quantities correlate with biological sample properties? Beyond quantifying the state of the light, quantum effects provided by twin photons can reach sensitivities only reached by quantum effects, such as in imaging, where the resolution is improved below the classical wavelength limit to the one specified by the deBroglie wavelength of both particles^10^; by two-photon imaging via fourth-order coherence^11^; or by squeezing or related methods that reduce the measured uncertainty below the quantum limit^12^.

Optical spectroscopy has been widely employed in brain research, and is the only technique for brain imaging with the resolution at micrometer or sub-micrometer scale. In the brain there are billions of neurons, astrocytes, glia cells, vessels, and axons that form many complex tree branches or spider cobwebs. The propagating photon is virtually absorbed to upper brain states, and coherently scattered forward mainly as a ballistic photon, propagating along in thin medium^4^,^6^. Tegmark^13^ measured that the decoherence effects in the complex tree of the brain are extremely fast, at the order of 10 zs to 10 as. The entangled photons at 802 nm propagating in brain can interact with H_2_O and hemoglobin (Hb) in the brain vessels and undergo virtual and real transitions^14^. Water has little absorption at 800 nm and the effects of Hb and HbO_2_ are the same so it helps the dressed ballistic photons from dephasing via scattering from the void regions in the overlapping neural trees. It is therefore hypothesized that the entanglement of a photon will be degraded when it passes through brain tissue, hence, measuring the polarization and coherence between this photon and its paired photon that not passing through any tissue/media opens the door for a new imaging technique in brain research.

The objective of this paper is to investigate the polarization change of two entangled photons as the light travels through rat brain tissue. We considered the simplest case that using twin polarization-entangled photons at 802 nm, with one photon traveling through brain tissue while the other twin photon traveling in free space to a detector. We measured the non-local correlations between the photons via quantum state tomography, and from them obtained various measures of quantum entanglement. We investigated to what degree the mixture induced by scattering depends on the depth of penetration into tissue media^15^. We found that despite scattering, brain tissue of various thickness shows strong photon entanglement, which is preserved through brain tissue layers up to 400 μm, and that the correlation with thickness is not as strong as with other parameters such as water content and type of tissue.

## Results

We have measured the preservation of entanglement as one photon of a pair passed through different types of tissue slices from rat: brain cortex, brain stem, and kidney. Before reaching the light collecting optics, the light went through polarization-state-projecting optical elements. On each arm we had the sequence of quarter-wave plate, half-wave plate, and fixed Glan-Thompson polarizer. These allowed the necessary projections to examine the joint state of the photons via quantum state tomography^16^,^17^.

**Figure 1:**
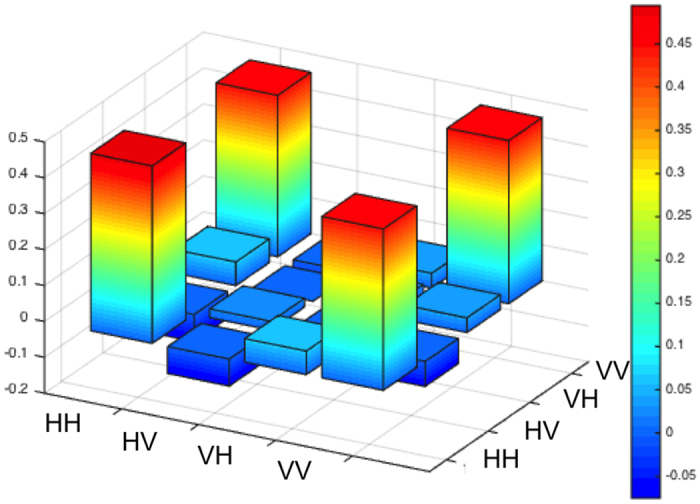
Histogram form of the density matrix of the light after passage through tissue with thickness 400 μm.

Figure 1 shows a typical experiment result of the real component of the density matrix of the light in rat brain cortex tissue with a thickness of 400 μm. The expectation is four pillars at the four corners with value 0.5 with zero pillars elsewhere. As can be seen the measured matrix agrees quite well with expectations. From the tomographic measurements of each sample we obtained various entanglement measures, such as fidelity (probability that the light is in state 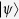 of Equation 1 in Methods), linear entropy (the degree of mixture; 0 for pure state and 1 for fully mixed state), and tangle (the degree of non-separability; 1 for a maximally-entangled Bell state and 0 for a product of mixed state). Our measurements were taken by integrating photon counts for times ranging between 10 s and 120 s, depending on the signal strength. The latter was dominated by the amount of scattering. The fidelity, linear entropy, and tangle for the case of Fig. 1 were respectively 0.957, 0.010(43) and 0.987(54). We further tested the entanglement by performing a Clauser-Horne-Shimony-Holt test of Bell’s inequalities using a sample with 40 μm thick, and obtained S = 2.76 ± 0.01 (S < 2 is expected from local-realistic hidden-variable theories, and 2.83 is expected of a pure maximally entangled state).

**Figure 2:**
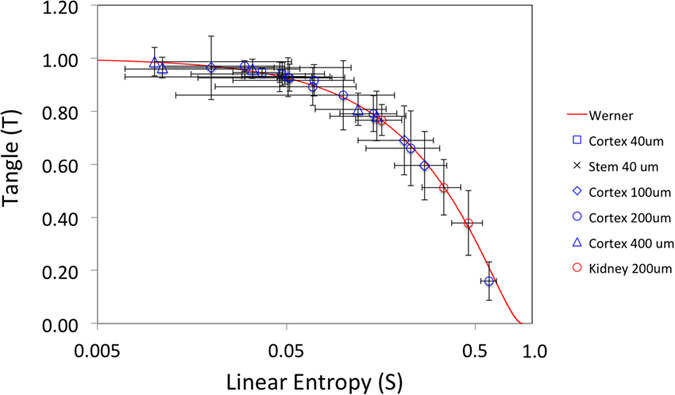
Entanglement measures (tangle and linear-entropy) of quantum state tomography of brain and kidney tissues. The symbols represent the various tissues used and their thicknesses (in micrometers). The solid line corresponds to the measures for Werner states.

Figure 2 is a tangle versus linear-entropy (TS) plot that illustrates the interplay between entanglement and mixture. It is plotted in a semi-log scale for a better examination of the data. The solid line represents the relation for Werner states^18^,^19^, that is, states where the state mixture is converted directly from the non-separable state. As can be seen, scattering degrades the entanglement along the Werner-state path, converting entangled photon pairs straight into incoherent pairs. The TS plot also shows stronger entanglement in brain tissue than in the kidney tissue. Entanglement was preserved irrespective of the thickness, depending more strongly on other sample characteristics, such as structure and degree of water in the sample. As time elapsed, the signal decreased, but followed the characteristic Werner-state curve in the TS plot. The latter is seen by the large spread in values for the samples with 200 μm.

Table 1 shows the complementary information, where the data corresponding to tissues with the same characteristics is averaged out. Because all data points fall on the Werner-state line, the averages do too. This is also seen in the small propagated error versus the much larger standard deviation. We found that although entanglement was degraded somewhat, it had no strong correlation with the thicknesses that we investigated. We note that the samples were fresh and still contained a significant amount of water. As time from preparation elapsed, the scattering increased and the entanglement decreased. When the samples were partially dry the scattering increased due to the increased number of scattering centers (air pockets), to levels that forced us to integrate significantly longer time. The entanglement dropped significantly after letting the sample dry for two hours. After 24 hours we were not able to detect any entanglement in tissues of thickness 100 μm and greater, suggesting that prepared samples may not retain their water contents for a considerable time. We also changed the apertures of the light entering the detector, but that did not change the entanglement measures significantly.

## Discussion

This study is the first investigation of correlation between entangled photons after propagation through rat brain tissue. It was demonstrated that the photon entanglement in polarization was preserved among brain tissue slices with different thickness. We focused on brain tissue because of its particular fabric (complex trees composing billions of neurons and axons and a lot of water) and the significance of optical techniques in brain research. The levels of entangled preservation found at large thicknesses encourage further differential studies of tissue (e.g., healthy versus unhealthy) for purposes of diagnosis. The use of photons at 802 nm was specially suited for this work to fit in tissue’s near-infrared optical window (650–950 nm).

The brain tissue with various thickness shows a strong entanglement - high T and low S in comparison to kidney tissue and aged tissues. This can be attributed to the unique structure of neuro network to channel the photon polarization and coherence in electromagnetic modes and quantum transfer pathways. The photon entanglement suggests quantum mechanics may be in operation of eigen pathways for photons’ passage through the brain’s special structure of neurons and axons. More research is needed to understand transport effects in brain tissue.

Work in the past on the passage of polarization entangled photons through inorganic scattering media found that the initial entanglement is degraded by multiple scattering, whereby the polarization degrees of freedom become coupled to the spatial degrees of freedom^20^. If the nature of the scattering is unitary (i.e., no absorption), the state of the light evolves from maximally entangled to Werner states^21^, where the degree of entanglement is degraded into a partial mixture of states and a maximally entangled Bell-state character^18^,^19^. With our particular entangled state we were able to violate Bell inequalities by more than 80 standard deviations. In our method of production of the entangled state, the state that we made is produced directly. To generate other Bell states we need to add wave plates to the output of the crystal. This would only decrease the fidelity of the state due to inherent imperfections of the optical devices. The use of other 3 states would in principle not improve our observation. We plan to do more measurements with other Bell states and for diseased brain tissue.

Some theories have been proposed about whether thought processes are mediated by quantum effects^22^, but the prevalent understanding, void of any actual measurements, is that it does not, because the quantum coherence that may exist within neurons is projected, or “measured” in the transfer of information from neuron to neuron via ion transfers^23^. For as far-fetched as these questions may be, only a deeper investigation of the role of quantum interactions in the brain will lead us closer to the answer.

## Methods

### Apparatus set up

**Figure 3:**
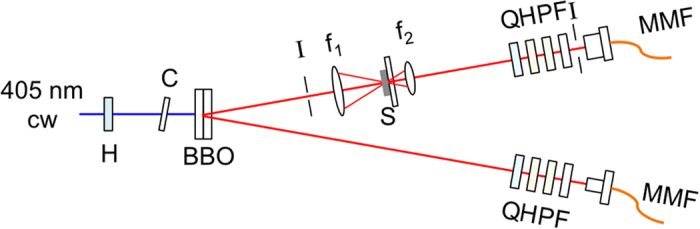
Apparatus used 401 nm linearly polarized light. State preparation optics included Half-wave plate (H), pre-compensating crystal (C) and pair of BBO crystals. Sample transmission optics included iris (I), lenses with focal lengths f_1_ and f_2_, and the sample (S). State projection optics included quarter-wave plates (Q), Glan-Thompson polarizers (P) and band-pass filters (F). Photon collection optics included small and large area collimators and multimode fibers (MMF).

A 50 mW beam from a diode laser of wavelength 401 nm was used to produce polarization entangled photon pairs via spontaneous parametric down conversion with two 0.5-mm thick type-I BBO crystals^24^. This prepared the pairs in the entangled state


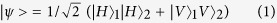


where H and V stand for horizontal and vertical states of linear polarizations respectively, and the subscripts refer to the two photons. The apparatus was arranged for channeling 802-nm photons with a bandwidth of 40 nm to avalanche–photodiode detectors through multimode fibers, as shown in Fig. 3. Detector signals were sent to photon counting and coincidence electronics. Before the collection optics were optical components to project the state of the light to any state of polarization. One photon (1) of a pair was sent through a tissue sample to the state projecting and collection optics, while the other photon (2) was sent straight to the state projection and collection optics. Photon 2 occupied a nearly 2-mm diameter spatial mode, equivalent to a Gaussian mode with a 0.3 mm beam spot.

**Figure 4:**
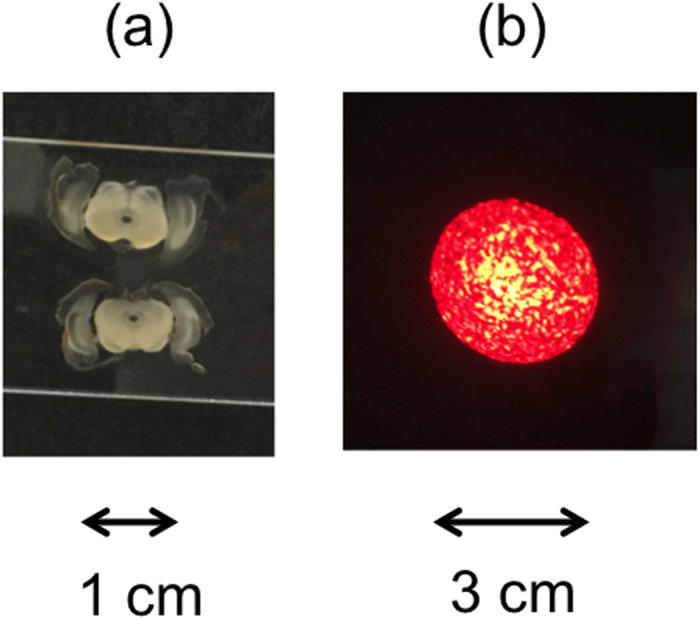
Photos of (**a**) two samples of brain tissue slices; and (**b**) of the speckle pattern created by a He-Ne laser beam with similar mode parameters as down-converted photons, mimicking the effect of the tissue sample on the focused light.

The light was focused onto a tissue sample deposited on the flat microscope glass slide. The Gaussian waist at the sample was about 86 μm, with a Rayleigh range of about 28 mm. Thus, the light going through the sample had nearly planar wavefronts. The unfocused light exhibited a speckle pattern, as shown in Fig. 4b for the case of a HeNe beam with similar mode parameters. We collimated the light pattern emerging from the sample and collected it with a large-area (11-mm diameter) fiber collimator preceded by an adjustable iris.

The density matrix of the quantum state of the light was obtained using quantum state tomography with numerical optimization^16^. The measures to quantify the quality of the quantum state: fidelity, linear entropy and tangle were calculated using standard methods^16^. The uncertainties in the linear entropy and tangle for each case were the standard deviation of 100 tomographies of Poisson-distributed data values centered about the measured values.

### Brain tissue sample preparation

Experimental procedures were in accordance with the guidelines and regulations approved by the Institutional Animal Care and Use Committee at the City College of the City University of New York. The protocol number is 918.2. The method used to prepare rodent brain tissue has been described in detail elsewhere^25^. A brief outline of the methods is given below with emphasis on the special features of the present experiments.

Rats were overdosed with an anesthetic. After the rat was completely anesthetized, decapitated, its brain were taken out and fixed overnight with 4% paraformaldehyde in 0.1 M phosphate buffer and then (PB) and subsequently immersed in 30% sucrose in 0.1 M PB for up to 48 hrs. Prior to slicing, the brain was quickly frozen and then sliced at thicknesses of 40, 100, 200 and 400 μm respectively by using a freezing stage microtome (American Optical, Buffalo, NY). The brain slices were then placed on coverslips at room temperature. The thicknesses of coverslips were 0.17 mm and the effect of thickness and its variation on tissue entanglement measurements were negligible.

## Additional Information

**How to cite this article**: Shi, L. *et al*. Photon Entanglement Through Brain Tissue. *Sci. Rep.* **6**, 37714; doi: 10.1038/srep37714 (2016).

**Publisher's note:** Springer Nature remains neutral with regard to jurisdictional claims in published maps and institutional affiliations.

## Figures and Tables

**Table 1:** Summary of the average data for different tissue types and thicknesses.

Tissue	Thickness (μm)	Fidelity	Linear Entropy	Tangle	Sample
Cortex	40	0.950 (1)	0.053 (10) [6]	0.924 (15) [10]	2
Stem	40	0.951 (4)	0.047 (12) [2]	0.931 (17) [1]	3
Cortex	100	0.89 (8)	0.17 (5) [11]	0.740 (65) [160]	4
Cortex	200	0.93 (9)	0.093 (13) [177]	0.868 (16) [254]	9
Kidney	200	0.84 (8)	0.32 (3) [15]	0.55 (5) [20]	3
Cortex	400	0.926 (23)	0.065 (17) [66]	0.900(22) [97]	5
None	0	0.954 (8)	0.050 (10) [6]	0.928 (15) [10]	3

Propagated uncertainties are within parenthesis, and standard deviations are within square brackets. Standard deviations that are larger than the propagated errors reflect that individual measurements scatter more due to other factors, such as time since preparation and amount of water in the tissue.
